# Ring-Screening to Control Endemic Transmission of *Taenia solium*

**DOI:** 10.1371/journal.pntd.0003125

**Published:** 2014-09-11

**Authors:** Seth E. O'Neal, Luz M. Moyano, Viterbo Ayvar, Silvia Rodriguez, Cesar Gavidia, Patricia P. Wilkins, Robert H. Gilman, Hector H. Garcia, Armando E. Gonzalez, for The Cysticercosis Working Group in Peru

**Affiliations:** 1 Department of Public Health and Preventive Medicine, Oregon Health & Science University, Portland, Oregon, United States of America; 2 Cysticercosis Elimination Program and Center for Global Health - Tumbes, Universidad Peruana Cayetano Heredia, Tumbes, Peru; 3 Instituto de Ciencias Neurológicas, Lima, Peru; 4 School of Veterinary Medicine, Universidad Nacional Mayor de San Marcos, Lima, Peru; 5 Division of Parasitic Diseases and Malaria, Centers for Disease Control, Atlanta, Georgia, United States of America; 6 Department of International Health, Bloomberg School of Public Health, Johns Hopkins University, Baltimore, Maryland, United States of America; 7 Department of Microbiology, School of Sciences, Universidad Peruana Cayetano Heredia, Lima, Peru; Ghent University, Belgium

## Abstract

**Background:**

*Taenia solium* is a major cause of preventable epilepsy in developing nations. Screening and treatment of human intestinal stage infection (taeniasis) within high-risk foci may reduce transmission and prevent epilepsy by limiting human exposure to infective eggs. We piloted a ring-strategy that involves screening and treatment for taeniasis among households located nearby pigs heavily-infected with the larval stage (cysticercosis). These pigs mark areas of increased transmission and can be identified by tongue examination.

**Methodology:**

We selected two villages in northern Peru for a controlled prospective interventional cohort pilot study. In the intervention village (1,058 residents) we examined the tongues of all pigs every 4 months for nodules characteristic of cysticercosis. We then screened all residents living within 100-meters of any tongue-positive pig using enzyme-linked immunosorbent assay to detect *Taenia* antigens in stool. Residents with taeniasis were treated with niclosamide. In both the intervention and control (753 residents) we measured incidence of exposure by sampling the pig population every 4 months for serum antibodies against cysticercosis using enzyme-linked immunoelectrotransfer blot.

**Principal Findings:**

Baseline seroincidence among pigs born during the study was 22.6 cases per 100 pigs per-month (95% confidence interval [CI] 17.0–30.0) in the intervention and 18.1 (95% CI 12.7–25.9) in the control. After one year we observed a 41% reduction in seroincidence in the intervention village compared to baseline (incidence rate ratio 0.59, 95% CI 0.41–0.87) while the seroincidence in the control village remained unchanged. At study end, the prevalence of taeniasis was nearly 4 times lower in the intervention than in the control (prevalence ratio 0.28, 95% CI 0.08–0.91).

**Conclusions/Significance:**

Ring-screening reduced transmission of *T. solium* in this pilot study and may provide an effective and practical approach for regions where resources are limited. However, this strategy requires validation in larger populations over a greater period of time.

## Author Summary

*Taenia solium* is a zoonotic parasite which infects humans and pigs resulting in the cysticercosis/taeniasis duo of neglected tropical diseases. It is commonly known as the pork tapeworm. Infection of the human brain with this parasite causes up to a third of epilepsy in Latin America, Asia and Africa. Infection of pigs is a food-safety issue, as consumption of contaminated pork results in intestinal tapeworm infection in humans who may then transmit the parasite to others. Pig infection also results in economic harm to small landowners who cannot sell the contaminated pork. Despite increasing worldwide attention to these diseases, sustainable and effective control strategies have been elusive. One possible approach is to direct limited screening and treatment resources to specific subpopulations which have the highest risk of infection. In this study we found that screening for intestinal tapeworms among people who live nearby a heavily-infected pig reduced transmission of the parasite in the overall community by 40% over one year. This practical strategy could be implemented as a potentially sustainable community-based intervention in the poor rural regions of world where the disease is endemic.

## Introduction

**Figure 1:**
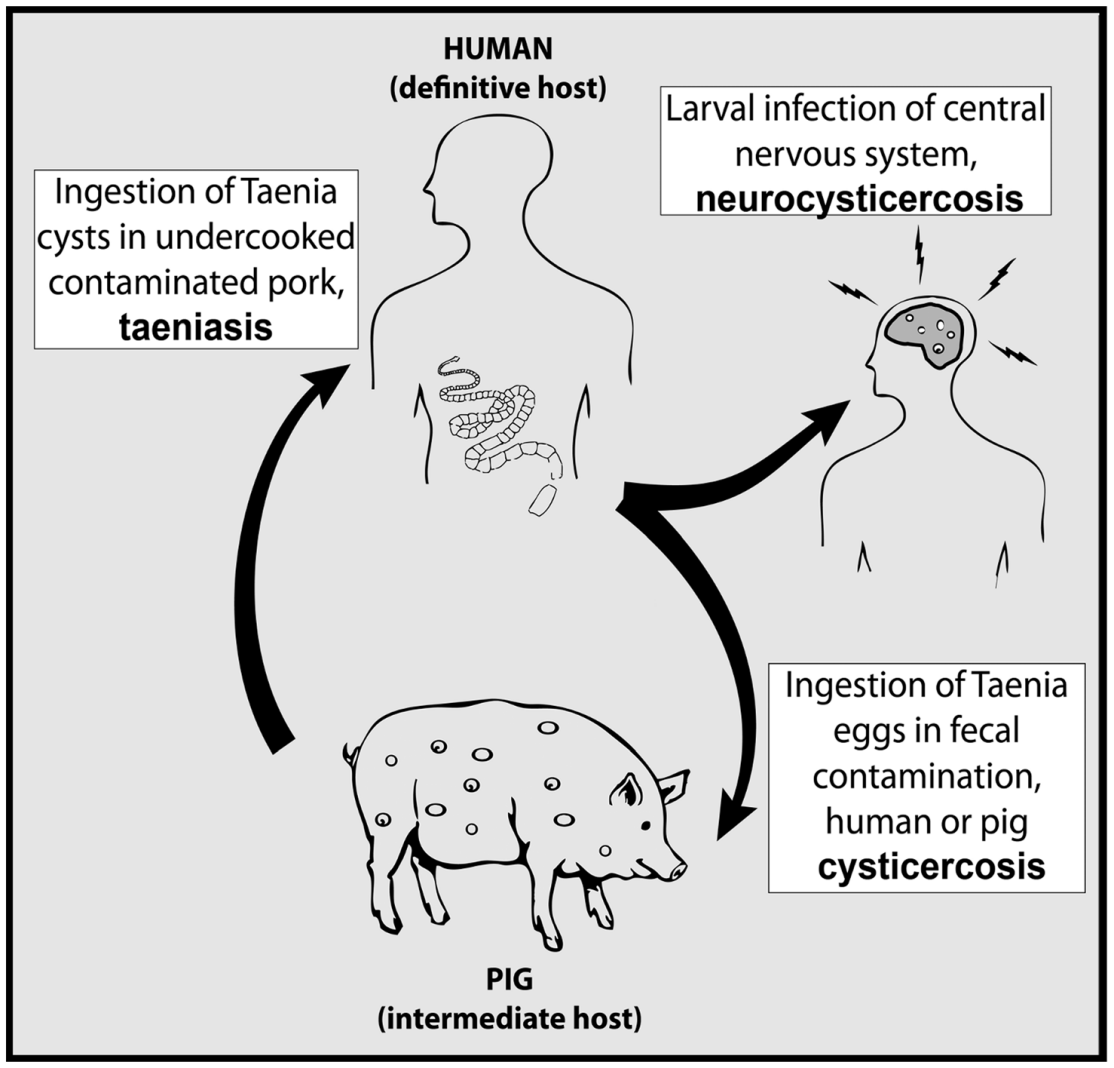
Lifecycle of *Taenia solium*.

*Taenia solium*, commonly known as the pork tapeworm, is a zoonotic parasite responsible for the cysticercosis/taeniasis duo of neglected tropical diseases. The reproductive lifecycle of this parasite is complex involving infective stages in both humans and pigs (Figure 1). Humans are the definitive host capable of harboring the adult egg-producing stage of the parasite in the intestine, a disease called taeniasis. People with taeniasis shed infective *T. solium* eggs in their feces. Pigs acquire the larval stage of infection in their tissues, a disease called cysticercosis, by consuming human feces containing *T. solium* eggs. Ingested tapeworm eggs release oncospheres that invade the intestinal wall and disseminate through the bloodstream to form cysts throughout the body. The lifecycle completes when a human consumes pork contaminated by *T. solium* larval cysts, as these may then develop into adult egg-producing intestinal tapeworms. This lifecycle occurs primarily in regions lacking sanitary infrastructure where pigs are allowed to roam and access raw sewage.

Humans can also acquire cysticercosis through incidental ingestion of tapeworm eggs. Neurocysticercosis (NCC) occurs when cysts develop within the central nervous system and may result in neurologic manifestations including seizure, headache, intracranial hypertension, encephalitis, cognitive impairment and stroke [1], [2]. Given the fecal-oral route of transmission and the lack of sanitary infrastructure in rural impoverished regions, exposure to the parasite is common. Brain lesions consistent with NCC can be found in 10–20% of residents in rural endemic villages [3]–[5]. In endemic areas around the world 30% of seizure disorders may be attributable to NCC [5]–[7]. In Latin America alone up to 1.35 million people have seizure disorders secondary to NCC [8], [9]. Control or elimination strategies for *T. solium* are needed to reduce the burden of neurologic disease in affected areas.

Treatment of taeniasis is an important component of control strategies, as this stage of infection is the direct cause of cysticercosis in both humans and pigs. However, identifying taeniasis in the community is difficult because people with adult-stage intestinal infection rarely have symptoms [10]. In addition, laboratory methods with adequate sensitivity for detecting taeniasis are not available in most endemic regions. Mass presumptive chemotherapy in a single round with niclosamide or praziquantel has been attempted to control transmission in multiple countries [11]–[14]. This strategy may temporarily decrease the prevalence of porcine cysticercosis and human taeniasis but transmission rapidly returns to baseline levels if underlying risk factors remain unchanged [14]. Incomplete participation with treatment, imperfect efficacy of single-dose regimens and migration of new tapeworm carriers into treated areas ensures that persistent cases of taeniasis can maintain transmission [15]. An additional drawback is that mass interventions may not be appropriate to control *T. solium* taeniasis given the relatively low prevalence (typically 2–3%) in endemic areas. Mass treatment in this scenario implies that the vast majority of treatment resources and associated risks are applied to those who do not need it.

An alternate approach involves focusing screening or treatment efforts on specific groups of people that have increased risk for taeniasis [16]. Selective treatment of these smaller high-risk populations can reduce the overall prevalence of infection while limiting the number of treatments administered and the frequency of adverse events [17]. This strategy is particularly effective when the target disease is highly clustered, as has been shown for *T. solium* in multiple countries [18], [19]. However, the experience of mass treatment demonstrates that control pressure must be sustained in the presence of persistent risk factors. Practical methods to identify specific high-risk foci within endemic communities are therefore needed for a targeted approach to be sustainable.

One possible method involves identifying people as at increased risk for taeniasis if they reside nearby a heavily-infected pig. Pigs that harbor between hundreds or thousands of larval cysts have been intensely exposed through repeated or massive ingestion of tapeworm eggs. It follows, therefore, that these heavily-infected animals might mark specific geographic areas within communities where taeniasis is present. This could provide a practical and potentially sustainable approach in resource-poor areas as pigs with a heavy-burden of cysts are easily identified during slaughter. They can also be identified while the pig is still alive by examining the tongue for visible or palpable nodules [20]. In many areas of Latin America tongue examination is an established and routine market practice that pig buyers use to avoid purchasing infected animals. However, evidence supporting this approach is very limited. In one rural Peruvian community the prevalence of taeniasis was >8 times higher (4/79 *vs.* 2/323) among residents living within 100 meters of a heavily-infected pig compared to residents living outside this range [21].

The objective of this study was to evaluate whether screening for taeniasis within a defined geographic radius around heavily-infected pigs (ring-screening), followed by treatment of identified carriers, can reduce transmission of *T. solium* in a rural endemic setting. If ring-screening proves both effective and practical, it could be implemented as a potentially sustainable control intervention for endemic areas.

## Methods

### Ethics statement

This study was reviewed and approved by the Institutional Review Boards at the Universidad Peruana Cayetano Heredia (UPCH) and at Oregon Health & Science University (OHSU). All adult participants provided written informed consent. Written informed consent from a parent or guardian was required for participation of minors. The study was also reviewed by the Institutional Ethics Committee for the Use of Animals at UPCH as well as the Institutional Animal Use and Care Committee at OHSU. Treatment of animals adhered to the Council for International Organizations of Medical Sciences (CIOMS) International Guiding Principles for Biomedical Research Involving Animals.

### Hypothesis

Screening for and treating taeniasis among households located in the immediate vicinity of a pig heavily-infected with cysticercosis will reduce transmission of *T. solium* in an endemic community.

### Study design and outcome measure

**Figure 2:**
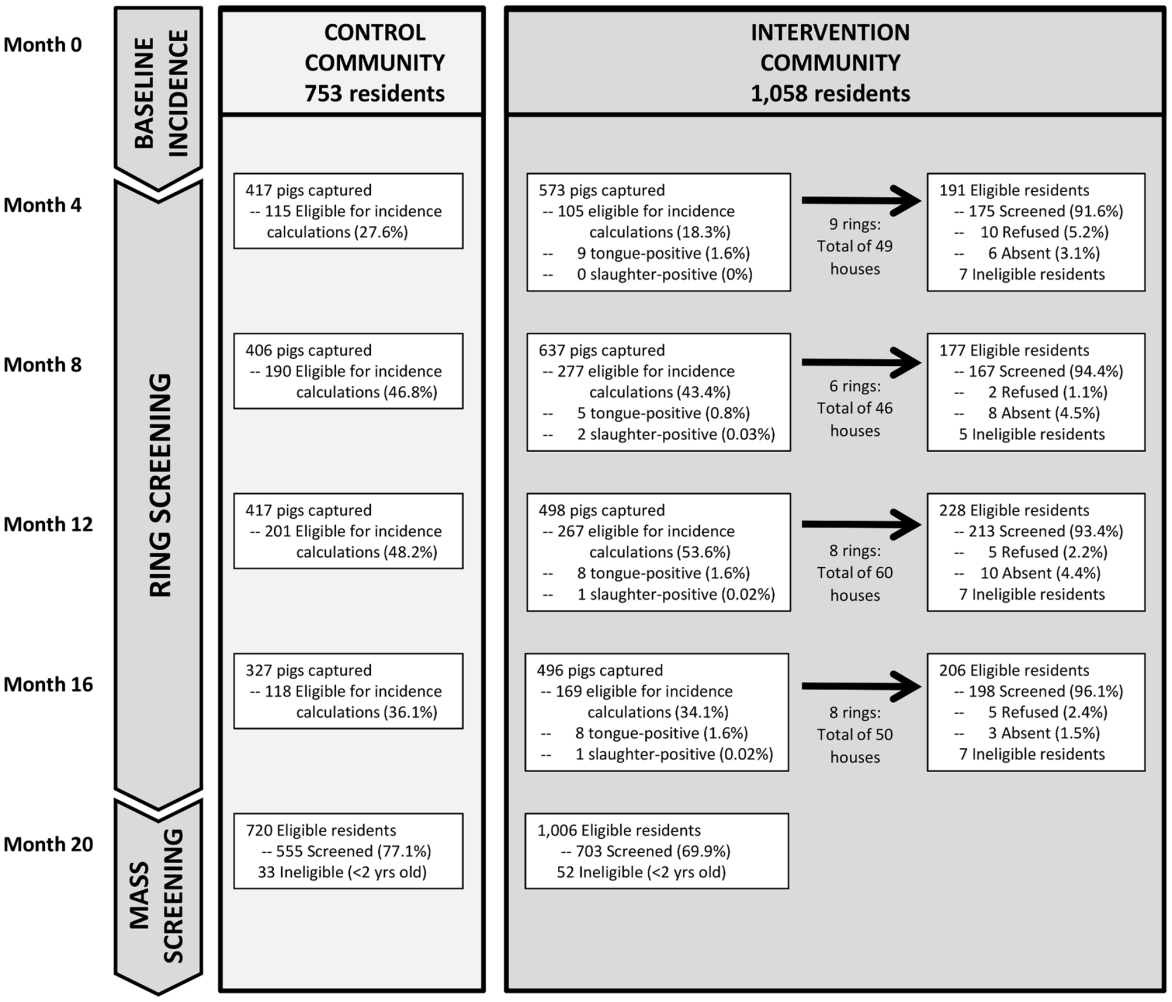
Flow diagram for community trial comparing ring-screening versus control.

This pilot study was a prospective interventional cohort involving two villages (intervention and control). In the intervention village only we implemented ring-screening to control parasite transmission (Figure 2). The primary outcome measure in both villages was the seroincidence among pigs born during the intervention period. Pigs raised for consumption in the rural villages are typically slaughtered in their first year of life. As the pig population rapidly turns over, new cohorts of unexposed pigs continuously arise in the community. Monitoring the seroincidence among pigs born during the intervention period therefore provides a time-sensitive indicator of the degree of parasite transmission. We used the enzyme-linked immunoelectrotransfer blot (EITB LLGP) to detect serum antibodies that indicate exposure to *T. solium* eggs [22].

### Study site and participants

We conducted this study in Piura, a Province in the northern coastal region of Peru where *T. solium* is known to be endemic [5], [23], [24]. In this arid agricultural region villagers frequently raise pigs as a source of income and meat protein. Pigs are typically allowed to roam unrestrained in the village to forage as this reduces or eliminates owner investment in feed. However, this practice also allows pigs to consume human feces as open defecation is common. While many households have corrals constructed of native materials, pigs are typically kept within them only for the few weeks prior to sale.

We selected two villages based on their similar size and terrain, and for the visible presence of unrestrained pigs. Surpampa (1,058 residents) received the ring-screening intervention and Santa Ana (753 residents) served as the control. These villages are separated by a distance of approximately 30 kilometers, which limited the opportunity for intermixing of their human or pig populations. We invited all residents to participate in the study, defining a resident as an individual who slept more than 2 nights per week in the village. Taeniasis is rare among children less than 2 years old so this group was excluded from screening activities although it was included in the census.

We began by mapping both villages, recording latitude and longitude coordinates of each house with global positioning system (GPS) receivers (GeoExplorer II; Trimble, Sunnyvale, CA) using post-processed differential correction for sub-meter accuracy. For each house in the intervention village, we pre-determined the subset of neighboring houses located within 100-meters radius using a geo-referenced map created with ArcMAP10 GIS software (ESRI; Redlands, CA). We chose the 100-meter radius as a familiar measurement which could be readily applied in a community-based control intervention. Previous work in this area demonstrated higher prevalence of taeniasis within this range [21]. We refer to these pre-determined clusters of houses as screening rings.

We then visited all houses in both villages to enroll participants. We collected household-level information on occupancy, presence of electricity, sanitary facilities, water source and animal husbandry practices, as well as individual-level data including age and sex of household members. Education was provided to all household members as a group at the time of enrollment as part of the consent process. This consisted of a brief discussion (∼5 minutes) of the *T. solium* lifecycle, methods of prevention and economic benefits of raising healthy animals. Simple lifecycle diagrams and pictures of the different stages of infection were shown to improve understanding.

### Determining seroincidence among pigs

Our veterinary teams returned to both villages in 4 successive sampling rounds (months 4, 8, 12 and 16) to capture all pigs, to tag them with a unique identifier and to collect a 5-ml blood sample from the *vena cava*. The identification tag allowed us to track individual pigs throughout the study. We excluded pregnant sows and piglets less than 6 weeks old from blood collection to avoid the possibility of harming these sensitive animals. Approximately 3–4 days per round were required to completely sample the pig population of both villages.

To determine the seroincidence of exposure, we followed a cohort of pigs that were between 6 weeks and 4 months old when first sampled. Pigs could enter this cohort during any sampling round. We followed the serologic results (EITB LLGP) of these pigs through subsequent sampling rounds until they seroconverted to positive status, were lost to follow-up or until study completion. Therefore, by the end of the study the cohort included pigs aged 6 weeks to 16 months old. Pigs that met age criteria and were seropositive when first sampled were considered to have seroconverted during that round. This overall strategy ensured that the analysis was based on a homogenous group of pigs that did not have previous environmental exposure to *T. solium* eggs. The seroincidence reported at each sampling point reflects the underlying risk of exposure during the preceding 4-month interval.

### Surveillance for heavily-infected pigs and ring-screening for taeniasis

We conducted surveillance for heavily-infected pigs in the intervention village only. During each serosampling round (months 4, 8, 12 and 16) our veterinary teams inspected the tongues of all captured pigs in the intervention village for nodules or cysts characteristic of *T. solium* cysticercosis. To examine the tongue, the animal was manually restrained and a hard wooden pallet was used to keep the mouth open. The veterinarian then gently retracted the tongue with a cloth visually inspecting and palpating the entire length of the underside of the tongue. The pig was considered tongue-positive if characteristic cysts or nodules were either seen or felt [20]. Villagers were also asked to report any pigs with cysts in the muscle tissue at time of slaughter at any point in the study period. Reports were made to the health post and a local study team member verified the infection. Both tongue-positive and slaughter-positive pigs were considered heavily-infected. We offered to purchase all heavily-infected pigs to ensure that contaminated meat would not be consumed.

When a heavily-infected pig was detected, we then tested for taeniasis among all residents living within the 100-meter ring surrounding the house where the heavily-infected pig was owned. Field teams visited all residents living within this ring, collecting a 5-ml blood sample via venipuncture in standard serology vacuum tubes and handing out a 500-ml plastic container with lid for collection of a whole stool sample. We provided soap and instructions on hygienic collection of stool to each resident. Teams returned to collect the stool samples within 24 hours.

### Case definition, treatment and follow-up of taeniasis

There is no standard case definition for taeniasis given the limitations of existing diagnostic tools. Stool microscopy has poor sensitivity as egg excretion in stool is intermittent. Enzyme-linked immunosorbent assay (ELISA) for *Taenia sp.* antigens in stool is highly sensitive but false-positive results may occur near the standard cutoff, presumably due to non-specific binding [25]. A recombinant enzyme-linked immunoelectrotransfer blot (EITB rES33) which detects serum antibodies is both highly sensitive and specific to *T. solium* taeniasis, but it cannot differentiate active from previously cleared infection [26]. Given these limitations, we categorized taeniasis as either suspected or confirmed according to the following case definitions:

#### Suspected taeniasis

Stool coproantigen ELISA result of optical density ratio (ODR)≥7.5%. The ODR for each sample was calculated as the ratio of the optical density (OD) value of the sample relative to the OD value of the strong positive *T. solium* control.

#### Confirmed taeniasis

Any suspected taeniasis in which *Taenia* species material (eggs, proglottids or scoleces) was present in initial or post-treatment stool samples *or* any stool coproantigen ELISA result of ODR≥40%

Serologic results of the EITB rES33 were evaluated for concurrence with stool results but were not used in the case definition. We definitively identified the species as *T. solium* when any of the following conditions were met; 1) DNA identification of *T. solium* by PCR restriction enzyme analysis (REA-PCR), 2) presence of 10 or fewer uterine branches in gravid proglottids or 3) presence of rostellar hooks on the scolex.

We treated participants with suspected or confirmed taeniasis with a single oral dose of niclosamide according to their weight (11–34 kg received 1 g; 35–50 kg received 1.5 g; >50 kg received 2 g). We collected a repeat stool sample two-weeks after treatment to verify cure. Those with persistent infection were re-treated with niclosamide and followed until the infection was cleared. Other pathogenic intestinal parasites found during stool screening were provided appropriate treatment through the local health center. The period between detection of a heavily-infected pig and treatment of any taeniasis found within the ring was approximately 3 weeks.

### Mass treatment and screening of humans for taeniasis

We returned to both villages at study completion (month 20) to conduct mass treatment and screening for taeniasis. We visited all residents in their homes and offered presumptive treatment with a single oral dose of niclosamide. Those who accepted treatment were given a 500-ml plastic container with lid and soap and were instructed to provide the first whole stool sample within 24 hours after treatment. We used this post-treatment screening strategy to increase the likelihood of detecting *Taenia sp.* material in those who were infected.

### Laboratory procedures

We examined stool samples macroscopically in a temporary field laboratory for the presence of *Taenia sp*. scoleces or proglottids. We placed 10 ml fecal aliquots in 40 ml of 5% formol-Phosphate Buffered Saline, pH 7.2 (PBS) in a sealed propylene tube. These samples were transported by ground at ambient temperature to the Center for Global Health laboratory (Tumbes, Peru) where they were concentrated by sedimentation and examined by light microscopy for the presence of *Taenia* sp. eggs. We then shipped the fecal samples by air to the CNS Parasitic Diseases Research Unit, Universidad Peruana Cayetano Heredia (Lima, Peru) for analysis. We used ELISA to detect *Taenia* sp. coproantigens as previously described [27], with the exception that the capture antibody and conjugate used were specific to *T. solium* [28]. We determined the species of taeniasis whenever possible, either by examination of scoleces or gravid proglottids or by PCR-restriction enzyme analysis as previously described [29].

Human and pig blood samples were stored chilled in ice coolers while in the field. They were then centrifuged in a field laboratory where 1.5 ml aliquots of sera were placed in microtubules and stored at −20°C. Frozen sera were then shipped by air to the CNS Parasitic Diseases Research Unit for further analysis. Pig sera were analyzed by enzyme-linked immunoelectrotransfer blot for presence of antibodies against *T. solium* cysts (EITB LLGP) as previously described [22]. The EITB LLGP assay uses an enriched fraction of homogenized *T. solium* cysts containing 7 *T. solium* glycoprotein antigens, GP50, GP42, GP24, GP21, GP18, GP14, GP13. Reaction to any of these 7 glycoprotein antigens is considered positive. We also analyzed human sera by EITB for presence of antibodies against recombinant antigens specific to *T. solium* adult stage infection (EITB rES33). The EITB rES33 is based on baculovirus expression system-purified recombinant antigen rES33 [26].

### Statistical methods

We analyzed all data in STATA SE12 (StataCorp; College Station, TX). We used Chi-square or Fisher's exact tests to compare distributions of proportions or to examine association between pairs of categorical measures. We used t-tests to compare means of continuous variables. All tests were 2-sided with significance set at 0.05. We used Poisson family Generalized Estimating Equations (GEE) with a log-link and robust sandwich-type errors to estimate the risk of exposure among pigs while accounting for the effect of intra-household clustering. We used quasilikelihood information criteria (QIC) to select the working correlation structure and the variables to include in the final model. Retained variables included those that decreased the QIC value relative to the saturated model. We included variables coding presence of household latrine or corral due to the presumed effects of these variables on transmission and the observed differences in these variables between villages. The outcome variable was the count of pig seroconversions to positive aggregated by sampling period and stratified by covariates including village (intervention or control), household, pig age in months, sex of pig, presence of corral and presence of latrine. The offset variable was the log of the total observed pig-months per strata. We report population-averaged seroincidence as the number of new seroconversions per 100 pigs per month during each 4-month sampling interval. Similarly, we used binomial family GEE with log-link and robust sandwich-type standard errors to estimate population-averaged prevalence of taeniasis at study completion between villages.

## Results

### Village characteristics and census data

Our study population included 1,811 people, including 1,058 (58.4%) people in the intervention community and 753 (41.6%) in the control community. There were 85 children 2 years old and younger who were ineligible for taeniasis screening based on age. There were some significant differences relevant to risk of *T. solium* transmission in the baseline characteristics of the two communities (Table 1). Specifically, while a similar proportion of households raised pigs in both communities, corrals were present more often in homes of pig owners in the control community than in the intervention community (83.9% vs. 52.1%. *p*<0.01). There were also more households with latrines in the control community compared to the intervention community (67.6% vs. 54.4%, *p*<0.01).

**Table 1:** Characteristics of participating households in intervention and control villages, Piura, Peru.

	Intervention community *n* = 217	Control community *n* = 185
Number of residents	1058	753
Single family dwellings, *n* (%)	189 (87.1)	168 (90.8)
Houses with latrines, *n* (%)	118 (54.4)	125 (67.6)
Houses with filtered water, *n* (%)	203 (93.5)	147 (79.5)
Houses with electric service, *n* (%)	183 (83.9)	155 (83.8)
Houses raising pigs, *n* (%)	144 (66.4)	112 (60.5)
Corral present on property, *n* (%)	75 (52.1)	94 (83.9)
Mean no. of pigs raised (SD*)	5.7 (4.9)	4.4 (5.1)
Mean no. of residents per house (SD*)	4.9 (2.3)	4.1 (1.9)
Mean no. of rooms per house (SD*)	4.1 (1.6)	3.6 (1.2)

* SD  =  standard deviation

### Surveillance for heavily-infected pigs and ring-screening for taeniasis

We captured and examined 2,410 individual pigs over the 4 rounds of the study, including 1,444 (59.9%) from the intervention village and 966 (40.1%) from the control (Figure 2). In all we identified 34 heavily-infected pigs, 30 (88.2%) of which were tongue-positive and 4 (11.8%) which were slaughter-positive. The majority of the tongue-positive pigs had positive confirmatory serology (29/30; 96.7%) indicating presence of antibodies against *T. solium* cysticercosis; 24 (80.0%) had 5–7 reactive bands on EITB LLGP, 4 (13.3) had 2–4 bands, one (3.3%) had a single band and one (3.3%) was negative. There were 3 instances in which two tongue-positive pigs were found in the same household during a single round.

We conducted screening for taeniasis within 31 rings in the intervention community over a 12-month intervention period (Figure 2). Within these 31 rings there were a total of 589 individuals representing 55.7% of the total population of the intervention village. The total population eligible for ring-screening (≥2 years old) was 576. Within the eligible population, 545 (94.6%) residents participated in screening; 369 (67.7%) were screened in a single round, 144 (26.4%) were screened in two rounds and 32 (5.9%) were screened in three rounds.

Of the 526 participants who provided a stool sample in any round, 35(6.7%) had suspected taeniasis. Two had suspected taeniasis in more than one round. Of the 35 suspected cases, 17 (48.6%) were subsequently confirmed to have taeniasis. The prevalence of confirmed taeniasis among participants screened in ring-screening was therefore 3.2% (17/526). Among the confirmed cases, 15(88.2%) were definitively identified as *T. solium* by PCR or by examination of scoleces/gravid proglottids, one (5.9%) was *T. saginata* (ODR = 21%, rES33 negative) and one (5.9%) could not be definitively identified as no parasite material was recovered (ODR = 111%, rES33 positive). Stool and serum screening results for each round are presented in Table 2.

**Table 2:** Results of ring-screening for taeniasis among residents within 100-meters of a pig heavily-infected with cysticerci.

	Round 1 no. (%)	Round 2 no. (%)	Round 3 no. (%)	Round 4 no. (%)	All rounds* no. (%)
Number eligible for screening	191	177	228	206	576
Number screened**	175 (91.6)	167 (94.4)	213 (93.4)	198 (96.1)	545 (94.6)
Provided stool sample	165 (94.3)	163 (97.6)	199 (93.3)	189 (95.5)	526 (96.5)
Suspected taeniasis (ODR†≥7.5%)	14 (8.5)	10 (6.1)	4 (2.0)	9 (4.8)	35 (6.7)
Confirmed taeniasis	5 (35.7)	7 (70.0)	2 (50.0)	3 (33.3)	17 (48.6)
ODR†≥40%	4 (80.0)	7 (100)	2 (100)	3 (100)	16 (94.2)
Stool microscopy positive	5 (100)	7 (100)	2 (100)	2 (66.7)	16 (94.2)
Unconfirmed taeniasis	9 (64.3)	3 (30.0)	2 (50.0)	6 (66.7)	18 (51.4)
Provided blood sample	158 (90.3)	155 (92.8)	189 (88.7)	179 (90.4)	514 (94.3)
Serum EITB rES33 positive	30 (19.0)	21 (13.6)	33 (17.5)	44 (25.6)	103 (20.0)

* Unique individuals only. Some individuals were tested in multiple rounds.

** Includes individuals who provided a sample of stool, blood or both.

† ODR  =  Optical density ratio, calculated as the ratio of the optical density (OD) value of the sample relative to the OD value of the strong positive *T. solium* control.

Both serum and stool were available for 498 participants including 33 with suspected taeniasis. Twenty (60.6%) of these 33 were also positive on EITB rES33. However, the prevalence of rES33 positivity was much higher (94.1%) among confirmed cases (Table 3).

**Table 3:** Seroprevalence of positive serology for antibodies against *Taenia solium* taeniasis (EITB rES33) by taeniasis status.

		rES33 positive	
	*n*	no. (%)	95% CI*
No taeniasis	465	82 (17.6)	14.1–21.1
Suspected taeniasis, unconfirmed	16	4 (25.0)	3.0–47.0
Confirmed taeniasis	17	16 (94.1)	82.6–100

*CI = confidence interval.

### Seroincidence of antibodies against cysticercosis in pigs

A total of 1,112 (46.1%) of all pigs were eligible for seroincidence calculations. Of these pigs, 657 (59.1%) were from the intervention villages while 455 (40.9%) were from the control. We collected 1,442 total blood samples as individual pigs were sometimes captured in multiple rounds; 836 (75.2%) pigs were captured only once, 230 (20.7%) were captured in two rounds, 38 (3.4%) in three rounds and 8 (0.7%) pigs were captured in all four rounds.

**Figure 3:**
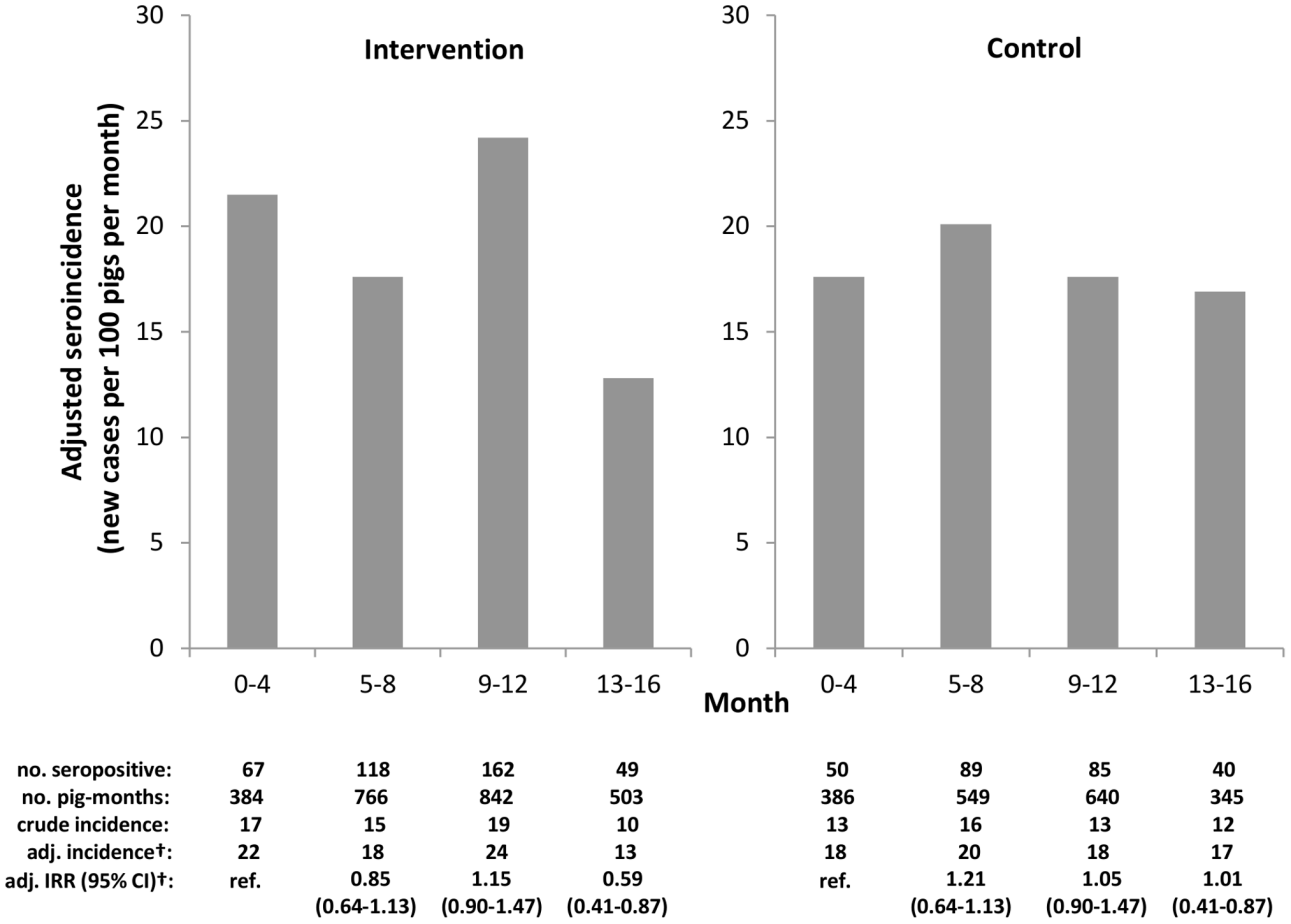
Seroincidence of antibodies against *Taenia solium* cysticercosis among pigs born during the study period. † Generalized estimating equations adjusted for household clustering, age and sex of pig, and presence of latrine or corral.

The baseline seroincidence in the intervention village was 22.6 new cases per 100 pigs per month (95% CI 17.0–30.0) compared to 18.1 cases per 100 pigs per month in the control (95% CI 12.7–25.9). There was substantial variability in seroincidence between periods in the intervention village that was not observed in the control. Figure 3 shows changes in seroincidence in both villages throughout the study period. Over the entire study period the seroincidence decreased 41% in the intervention community (incidence rate ratio [IRR] 0.59, 95% CI 0.41–0.87) and remained unchanged in the control village (IRR 1.01, 95% CI 0.70–1.47). There was 41% greater reduction in seroincidence between baseline and study end in the intervention village compared to the control village (IRR 0.59, 95% CI 0.35–0.97). Within the intervention village, there was a 51% reduction in seroincidence in the population of pigs raised in houses that fell within one of the screening rings (IRR 0.49, 95% CI 0.31–0.76). The estimated 31% seroincidence reduction in pigs raised in houses that were not within a screening ring was not statistically significant (IRR 0.70, 95% CI 0.36–1.35).

### Mass treatment and screening for human taeniasis

We collected post-treatment stool samples from a total of 1,258 (72.9%) villagers older than 2 years of age at study end (month 20). Participation in stool collection was higher during targeted ring-screening (526/576, 91.3%) than during mass screening in either the intervention (703/1,006; 69.9%; *p*<0.01) or the control villages (555/720; 77.1%; *p*<0.01). The adjusted prevalence of confirmed taeniasis was nearly 4 times lower in the intervention village than in the control at study end (PR 0.28, 95% CI 0.08–0.91). Similarly, the adjusted prevalence of suspected taeniasis was also lower in the intervention village (PR 0.44, 95% CI 0.18–1.08). The crude and adjusted prevalence ratios are presented in Table 4.

**Table 4:** Prevalence of taeniasis during mass stool screening at study end by eligibility for ring-screening intervention.

	no.	Suspected taeniasis no. (%)	adj. PR* (95% CI)	*p*	Confirmed taeniasis no.(%)	adj. PR* (95% CI)	*p*
Control village	555	14 (2.5)	ref.	–	9 (1.6)	ref.	–
Intervention village	703	10 (1.4)	0.44 (0.18–1.08)	0.07	5 (0.7)	0.28 (0.08–0.91)	0.03
Eligible for ring-screening	418	3 (0.7)	0.21 (0.06–0.80)	0.02	1 (0.2)	0.09 (0.01–0.73)	0.02
Ineligible for ring-screening	285	7 (2.5)	0.81 (0.32–2.01)	0.64	4 (1.4)	0.60 (0.17–2.08)	0.42

* adj. PR  =  Prevalence ratio adjusted for age, sex, number of household residents and household clustering using binomial family Generalized Estimating Equations with log link and robust sandwich-type errors.

## Discussion

This study provides initial evidence that ring-screening for taeniasis may reduce transmission of *T. solium* in a rural endemic area. By focusing screening for taeniasis on people living within 100-meters of a heavily-infected pig, and providing treatment only to those who were found to be infected, we observed >40% reduction in pig seroincidence over one year in the intervention village while the seroincidence in the control village remained unchanged. However, we also observed considerable variation in seroincidence in the intervention village which suggests that validation of this strategy in larger studies with longer follow-up time is necessary. Overall, we identified 35 people with suspected taeniasis and 34 heavily-infected pigs using this strategy.

To evaluate our intervention, we monitored the seroincidence in the pig population using EITB LLGP which detects the presence of circulating IgG antibodies against purified *T. solium* metacestode antigens [22]. It is important to recognize that a positive result on the EITB LLGP does not necessarily indicate active infection [30]. A positive result can occur from exposure to *T. solium* eggs that did not result in established infection, from previously established infection which has been cleared or by transmission of maternal antibodies in colostrum [31], [32]. Seroincidence results based on the EITB LLGP should therefore be interpreted broadly as a measure of overall exposure to *T. solium* eggs among the pig population. The EITB LLGP is an excellent assay for this purpose given that no cross-reactions have been reported [20].

Maternal transmission of antibodies rather than new antigen exposure likely contributed to the observed seroincidence in this study given that we measured seroincidence in young pigs. However, maternal transmission does not alter our conclusion that ring-screening was effective. Any sow that passed antibodies to its offspring in our study was exposed either during or prior to the intervention period. If the sow was exposed during the intervention, then the maternally-transmitted antibodies in the offspring are correctly interpreted as evidence of ongoing antigen exposure among pigs. On the other hand, if the sow was exposed prior to our intervention, then maternally-transmitted antibodies in the offspring erroneously suggest recent antigen exposure. Therefore, maternal transmission can only result in an underestimate of the true intervention effect.

The considerable variability in the observed seroincidence in the intervention village, including the temporary increase after 8 months, remains unexplained. This variability does not appear to be related to normal seasonal change given the stable seroincidence in the control village which lies in the same region and was measured concurrently. We controlled for the presence of latrines and corrals in our model which should have mitigated any interaction between seasonality and these factors. It remains possible that the difference is due to some unmeasured factor that varied between the villages. However, we suspect that the observed variability in the intervention village is the result of an endemically stable system reacting to the intervention itself. Fluctuation in pig seroprevalence has been noted in other studies after mass treatment for taeniasis [14], [33]. Longer-term follow up of this intervention would improve interpretation of the observed variability, as would longitudinal studies evaluating *T. solium* transmission dynamics in both stable and intervened settings.

The lower prevalence of confirmed taeniasis in the intervention community at study completion compared to the control community (nearly 4 times lower) provides additional supporting evidence that ring-screening was effective. This difference was statistically significant despite the fact that the study was not powered to measure this secondary outcome. We were unable to evaluate the change in taeniasis prevalence from start to end within each community as baseline mass-screening and treatment would have confounded the effect of ring-screening. Only one person with taeniasis was found at study end among people who had been eligible for ring-screening. Interestingly, while this person had negative stool findings when evaluated in the second round of ring-screening, they were positive for serum antibodies against *T. solium* taeniasis. This may reflect a false-negative stool result in a newly-acquired immature tapeworm. It should also be noted that the prevalence of tongue-positive pigs in the intervention village remained essentially unchanged throughout the study, suggesting continued transmission around persistent tapeworm carriers.

Participation is crucial for control strategies based on treatment of taeniasis. The low steady-state prevalence and long-lifespan of intestinal infection suggests that endemic transmission can be maintained by a small number of people with taeniasis [15]. A single person with taeniasis may shed tens of millions of potentially infectious eggs into the environment during the course of their infection. Overall participation was <75% in the single round of mass screening and treatment in this study, which left a large unscreened population in which taeniasis could persist. Participation in repeated mass treatment campaigns would likely only diminish over time. In contrast, participation in ring-screening remained >90% over several intervention rounds in this study.

The excellent participation in ring-screening may be due to the fact that it is a targeted approach in contrast to an intervention such as mass screening that is uniformly applied. The perception of increased risk may promote participation of people living nearby heavily-infected pigs. Ring-screening inherently reinforces the relationship between intestinal tapeworm infection in humans and cysticercosis in pigs. Triggered screening of humans in neighborhoods where visibly-infected pigs are found stresses to villagers that these two life stages of the parasite are connected. Lack of understanding of the parasite lifecycle is common in rural communities and contributes to resistance to risk-reduction measures such as corral and latrine use. Although minimal emphasis was placed on education in this study, improved knowledge likely contributed to the control gains observed in the intervention village.

Refinements to the screening and intervention methods used in this study could improve the overall effectiveness of this ring-strategy. The most intuitive would be to include treatment with oxfendazole for cysticercosis in all pigs raised within the rings. Additional control pressure applied to the larval stage of infection, particularly in high-risk areas, could further reduce the risk of taeniasis by limiting the supply of contaminated meat. Another possibility includes using other methods to identify the heavily-infected pigs and associated screening rings, such as ultrasound or serology which may be more sensitive. Not all pigs with heavy-exposure to a tapeworm carrier will have visible or palpable cysts on the tongue. Imperfect sensitivity of the tongue-test may account for the cases of taeniasis at study end among residents of the intervention village who were not part of a screening ring. However, use of more sensitive methods could generate an unmanageably large population requiring intervention in areas where transmission is high. It may be more practical to reserve highly sensitive methods for areas with low transmission or for when control gains using tongue-examination have reached plateau.

Ultimately, our goal is to develop control strategies for *T. solium* which are both effective and practical for rural resource-limited regions where this disease is endemic. Ring-strategy is particularly attractive because it lends itself to a community-based approach that is potentially sustainable. Surveillance based on tongue examination or presence of cysts in a slaughtered animal could be conducted by villagers themselves, although establishing a local system for reporting and intervention would be required. Furthermore, although our intervention included laboratory screening, empiric treatment without screening within the rings is an attractive alternative given the safety profile of niclosamide and the added benefit of eliminating any risk of false-negative screening. Empiric treatment with niclosamide for taeniasis and oxfendazole for pigs could be administered by trained community health workers. This could provide a low-cost alternative for regions which do not have the resources and infrastructure required for laboratory screening. Ring-strategy could be applied alone or in combination with other control interventions such as education, corralling or improved sanitation. It could also be applied after initial mass-treatment. Finally, it could also potentially be applied as a surveillance system for re-introduction of *T. solium* into regions where the parasite has been eliminated. For a comprehensive review of other control interventions, we refer readers to a 2009 World Health Organization report [34].

### Limitations

This pilot study has some important limitations which should be considered when interpreting the results. This was a non-randomized trial limited to two villages so allocation bias cannot be excluded. Corral and latrines were in fact significantly more common in the control village where the estimated baseline seroincidence was ∼25% lower than in the intervention. However, participant enrollment and baseline pig sampling was complete before these differences were detectable. We cannot predict whether the same degree of effectiveness would have been achieved if allocation had been reversed. The trial was also conducted in a region that has high endemic transmission of *T. solium*. Our results may not be generalizable to other regions of the world where the burden of transmission and underlying risk factors may not be the same. In particular, local variations in animal husbandry practices, sanitation infrastructure, housing density, topography, climate and participation could affect intervention results. Finally, the follow-up period was also limited in duration. While it is encouraging that significant reduction in seroincidence occurred in the intervention village only, long-term follow-up is needed to ensure that control gains are not temporary.

### Recommendations for future studies

Future studies to validate the effectiveness of ring-strategy should ideally include multiple sites, random allocation, longer intervention and follow-up periods and a larger sample size. The effectiveness and acceptability of empiric treatment of both humans and pigs within high-risk rings should be explored further, as should the viability of a community-based approach. In addition, direct comparison with periodic mass treatment or other control strategies is needed, including evaluation of cost, participation and other variables relevant to implementation.
